# Discrete Cosine Transform for the Analysis of Essential Tremor

**DOI:** 10.3389/fphys.2018.01947

**Published:** 2019-01-17

**Authors:** Jordi Solé-Casals, Iker Anchustegui-Echearte, Pere Marti-Puig, Pilar M. Calvo, Alberto Bergareche, José Ignacio Sánchez-Méndez, Karmele Lopez-de-Ipina

**Affiliations:** ^1^Data and Signal Processing Research Group, University of Vic-Central University of Catalonia, Barcelona, Spain; ^2^Department of Psychiatry, University of Cambridge, Cambridge, United Kingdom; ^3^Seidor Labs, Tona, Spain; ^4^EleKin Research Group, System Engineering and Automation Department, University of the Basque Country UPV/EHU, Donostia, Spain; ^5^Neurodegenerative Disorders Area, Biodonostia Health Research Institute, San Sebastián, Spain; ^6^Movement Disorders Unit, Department of Neurology, University Hospital Donostia, San Sebastián, Spain; ^7^Biomedical Research Networking Centre Consortium for the area of Neurodegenerative Diseases (CIBERNED), Madrid, Spain

**Keywords:** essential tremor, automatic drawing analysis, archimedes' spiral, discrete cosine features, automatic feature selection

## Abstract

Essential tremor (ET) is the most common movement disorder. In fact, its prevalence is about 20 times higher than that of Parkinson's disease. In addition, studies have shown that a high percentage of cases, between 50 and 70%, are estimated to be of genetic origin. The gold standard test for diagnosis, monitoring and to differentiate between both pathologies is based on the drawing of the Archimedes' spiral. Our major challenge is to develop the simplest system able to correctly classify Archimedes' spirals, therefore we will exclusively use the information of the *x* and *y* coordinates. This is the minimum information provided by any digitizing device. We explore the use of features from drawings related to the Discrete Cosine Transform as part of a wider cross-study for the diagnosis of essential tremor held at Biodonostia. We compare the performance of these features against other classic and already analyzed ones. We outperform previous results using a very simple system and a reduced set of features. Because the system is simple, it will be possible to implement it in a portable device (microcontroller), which will receive the *x* and *y* coordinates and will issue the classification result. This can be done in real time, and therefore without needing any extra job from the medical team. In future works these new drawing-biomarkers will be integrated with the ones obtained in the previous Biodonostia study. Undoubtedly, the use of this technology and user-friendly tools based on indirect measures could provide remarkable social and economic benefits.

## Introduction

Essential tremor is a neurological disorder 20 times more common than Parkinson's disease that affects individuals worldwide with a prevalence in the western world of about 0.3–4%. With regard to epidemiological analysis, the incidence of ET increases with age, both men and women are affected more or less equally, with an incidence of 23.7 per 100,000 people per year, and may also appear in children. In this scenario, studies suggest that the prevalence among elderly ranges between 3.9 and 14.0%. Moreover, 50 to 70% of essential tremor cases are estimated to be of genetic origin [1] and in these cases an early development of symptoms could appear. In the characterization of this disorder, ET is considered a kinetic rhythmic tremor (4–12 Hz) that only occurs when the affected muscle is exerting an effort, and its amplitude is variable with respect to age, but there is no gender predilection.

The risk of Parkinson's disease in people with essential tremor is higher than in the general population, and stress and fatigue may worsen the tremor. In addition, Parkinson's disease and essential tremor can also occur simultaneously and may appear in individuals of the same family. As far as symptoms of essential tremor are concerned, as in Parkinson's disease, tremor of the hand predominates and occurs in most cases, followed by or at the same time that tremor of the head, voice, neck, face, leg, tongue, trunk and walking difficulties (Louis and Vonsattel, 2007). The symptoms of ET produce a dramatic decline in the performance of daily activities and may lead to disabilities.

Therefore, early treatment of the disorder is essential in order to control and alleviate symptoms and increase the patients' quality of life. In recent years, significant progress has been made in the development of reliable and robust clinical biomarkers. However, despite their utility, some of the tests can be very invasive or involve high cost and technological requirements that make it impossible to apply them to all patients with motor disorders, especially when continuous monitoring is necessary. For these cases, new intelligent non-invasive diagnostic techniques have been developed based on indirect biosignals such as speech, writing or drawing. These developments can become valuable tools for early detection of disorders and friendly monitoring. Additionally, these techniques supervised by health specialists are managed by non-technical staff in the patient's usual environments without introducing stress or altering or blocking their abilities. The systems are very low cost and do not require extensive infrastructures or the availability of medical equipments. The biosignals obtained are simple, natural and easy to process and manage, and the tools are capable of producing information easily, quickly and economically (Lopez-de-Ipiña et al., 2013a,b; Zanuy et al., 2013; Laske et al., 2015). The literature and clinical practice establish that handwritten tasks can be used for the diagnosis of essential tremor and in this sense Archimedes' spiral is the reference test in clinical diagnosis (Pullman, 1998).

In the past, handwriting analysis was performed using an offline test without technological tools. In fact, only the writing or drawing itself (lines on paper) and the perception of the health specialist were available and analyzed. Nowadays, modern capture devices (digitizing tablets and pens) can gather dynamic data with their temporal dimension to include the evolution of the performance and quantitative measurements. Then, the analysis is carried out online with the available spatiotemporal information. The first papers published using digitizing tablets dates back to the 1990s (see Elble et al., 1996; Cameron Riviere et al., 1997; Pullman, 1998 for example), and spread significantly from this century (see Miralles et al., 2006; Zeuner et al., 2007; Haubenberger et al., 2011; Louis et al., 2012 for example), when new and more powerful tablets appeared on the market. These modern digitizing tablets collect not only the *x* and *y* coordinate points that describe the hand movement and the evolution of the pattern as it changes position, but can also collect other interesting features, such as the pressure exerted on the writing surface, the azimuth, the angles of the pen with regard to the vertical and horizontal axis, the altitude (Likforman-Sulem et al., 2017), as well as the movement in the air when there is no contact nor pressure between the pen and the paper or device (Sesa-Nogueras et al., 2012). This provides the possibility to analyse both the static characteristics and the dynamics of their evolution (Faundez-Zanuy, 2007):

- Static*:* Also known as “off-line” analysis. In these tests users write their handwriting/drawing on paper and afterwards the strokes are digitized through a camera or an optical scanner. Then, a biometric analysis is carried out.
- Dynamic*:* Also known as “on-line” analysis. In these tests, users write in a digitizing device, which acquires the drawing/handwriting in real time with the whole set of features abovementioned. Not only the strokes but also the spatiotemporal information is available and used.

The present work belongs to a larger cross-sectional study for the characterization of ET by indirect measures, and it is included in the general transversal study conducted at the Biodonostia Health Institute, which focuses on the characterization of genetic ET and is based on families with identified genetic loci. For the detection of ET, Archimedes' spiral has been selected as the reference test for the selection of linear and/or non-linear biomarkers from drawings and writing, bearing in mind that irregularities due to stress may also appear in control persons and patients with ET. A previous work that used the same data (but with other features and classification systems) can be found in (Lopez De Ipina et al. (2015). The main goal of the study is to analyse the capability of a classification system using exclusively the *x* and *y* coordinate points of the drawings. This is because we would like to use the handwriting exercise in real time using a tablet or phablet. In the next sections we detail the new proposed features obtained through the discrete cosine transform. Then, several automatic analysis systems, Linear Discriminant Analysis (LDA), k-Nearest Neighborhood (KNN) and Support Vector Machines (SVM), will measure the quality of the selected features. Obtained results will be compared with already available results with the same database in order to check the potential use of these new descriptors.

## Materials and Methods

### Acquisition System

The acquisition system is a digitizing tablet, the Intuos WACOM 4 2017, which is connected to a laptop trough a USB port and captures the spatial coordinates, the azimuth and altitude angles of the pen on the tablet, and the pressure exerted for it on the surface. Sampling frequency is set to 100 Hz. From this data we could infer other variables such as acceleration, speed, etc. (Jain et al., 1999; Sadikov Groznik et al., 2014).

### Database

**Figure 1:**
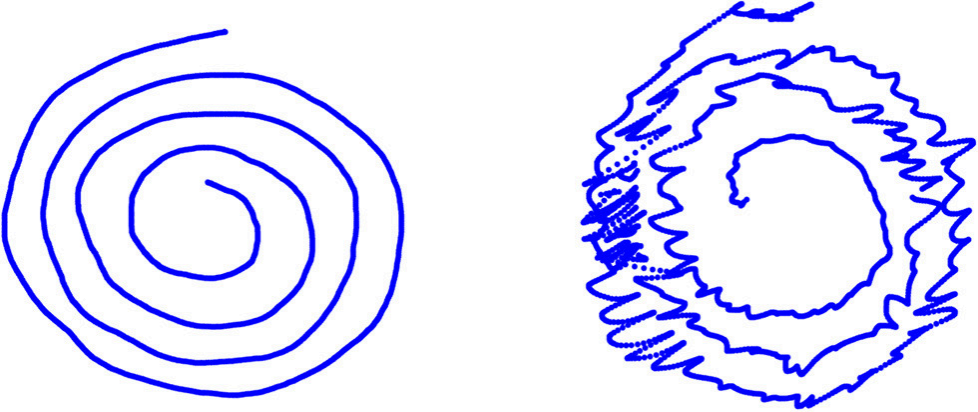
Example of the original drawing of Archimedes' spiral, performed by a control individual (**Left**) and an individual with essential tremor (**Right**).

In this paper we use the database named BIODARW, first presented in (Lopez De Ipina et al., 2015; López-de-Ipiña et al., 2016). We have a total of 21 control people (CP) and 29 ET people. The test consists of, among other exercises, drawing the Archimedes' spiral (Figure 1) with both the dominant and non-dominant hands. Therefore, originally the database contains 100 handwriting samples. In order to compare our results with the ones in Lopez De Ipina et al. (2015) and López-de-Ipiña et al. (2016), we will proceed as done in these works and will only use the BIODARWO subset, which consists of 51 samples: 24 samples for the ET group and 27 samples for the control group. The selection of these samples was as follows:

a. For the ET group, only the sample with the best quality is chosen (one hand), but 5 subjects are discarded due to the poor quality of the samples.
b. For the control group, the best sample (habitually the dominant hand) is kept, but in 6 cases, also the non-dominant hand is included

The medical team carried out this selection. Detailed information of recruitment acquisition procedure and selection of this subset can be found in López-de-Ipiña,(2016). Table 1 summarizes the features of the group with ET with regard to test features, diagnosis and demography. Due to lack of space, only the first 9 subjects are presented.

**Table 1:** Some examples of the database, together with electrophysiological test features and diagnosis using Fahn–Tolosa–Marin (FTM) scale values for the selected individuals with ET (ET_*x*).

**ET_*x***	**Electrophysiological test features**			**Diagnosis**	**Demography**	
	**Frequency (Hz)**	**Amplitude (V)**	**Pattern**	**FTM Scale**	**Age**	**Gender**
ET_01	8.5	20	Synchronous	1	48	Female
ET_02	6.5	variable	Alternating	8	72	Male
ET_03	10.5	200	Synchronous	1	46	Male
ET_04	4.5	503.6	Synchronous	3	80	Female
ET_05	6.6	298	Synchronous	22	68	Female
ET_06	9.5	46	Synchronous	2	46	Female
ET_07	5	173	Synchronous	50	75	Male
ET_08	6.5	159	Synchronous	40	75	Male
ET_09	8	128	Synchronous	9	75	Female

### Discrete Cosine Transform of Type II, Partial Reconstructions and Residues

Considering the set of N points *x*_*n*_ where *n* goes from 0 to *N* − 1, and N transformed coefficients *X*_*k*_, where *k* goes also from 0 to *N* − 1, the forward and backward expressions of the type II Discrete cosine transform take the form:

(1) $$\begin{array}{l} X_{k}=\overset{N-1}{\underset{n=0}{\sum }}c_{k}x_{n}\cos\left(\frac{\pi }{N}\left(n+\frac{1}{2}\right)k\right);\;k=0,\cdots \;,N-1 \end{array}$$

and,

(2) $$\begin{array}{l} x_{n}=\overset{N-1}{\underset{k=0}{\sum }}c_{k}X_{k}\cos\left(\frac{\pi }{N}\left(n+\frac{1}{2}\right)k\right);\;n=0,\cdots \;,N-1 \end{array}$$

and, Where *c*_*k*_ is defined as:

(3) $$c_{k}=\{\begin{array}{c} \sqrt{\frac{1}{N}};\;k=0\\ \sqrt{\frac{2}{N}};\;k\neq 0 \end{array}$$

Equations (1) and (2) show that, from all the coefficients *X*_*k*_, the N samples of the original *x*_*n*_ sequence is perfectly recovered.

Let us consider only the first L coefficients of *X*_*k*_ to reconstruct the original sequence *x*_*n*_, in order to obtain an approximation ${\tilde{x}}_{n}$ as follows:

(4) $$\begin{array}{l} {\tilde{x}}_{n}=\overset{L-1}{\underset{k=0}{\sum }}c_{k}X_{k}\cos\left(\frac{\pi }{N}\left(n+\frac{1}{2}\right)k\right);\;n=0,\cdots \;,N-1\;and\;\left(L<N\right) \end{array}$$

And the remaining *X*_*k*_, to form the residue ${\hat{x}}_{n}$ as:

(5) $$\begin{array}{l} {\hat{x}}_{n}=\overset{N-1}{\underset{k=L}{\sum }}c_{k}X_{k}\cos\left(\frac{\pi }{N}\left(n+\frac{1}{2}\right)k\right);\;n=0,\cdots \;,N-1 \end{array}$$

It comes directly from (4) and (5) that the original sequence *x*_*n*_ is ${x_{n}=\tilde{x}}_{n}+{\hat{x}}_{n}$. As commonly L≪N, the calculus of ${\tilde{x}}_{n}\;$involves fewer coefficients than the one for ${\hat{x}}_{n}$, therefore the residue ${\hat{x}}_{n}\;$is obtained more efficiently from ${\tilde{x}}_{n}\;$ as:

(6) $$\begin{array}{l} {\hat{x}}_{n}{=x_{n}-\tilde{x}}_{n} \end{array}$$

We propose the use of the DCT because this transformation is often used in lossy data compression applications. The property of the DCT that makes it suitable for compression is its high degree of spectral compaction; this means that the DCT representation of a signal tends to concentrate more of its energy in a small number of coefficients, the first ones, compared to other transformations such as DFT. Therefore, this characteristics will allow us to keep a small number of coefficients containing the fundamental information about the drawings.

### Extracted Features

The digitalizing tablet used was an Intuos Wacom 4. The pen tablet captures the spatial coordinates (*x*_*n*_, *y*_*n*_), the pressure, and the azimuth and altitude angles of drawing. In this study only the spatial coordinates (*x*_*n*_, *y*_*n*_) were used.

**Figure 2:**
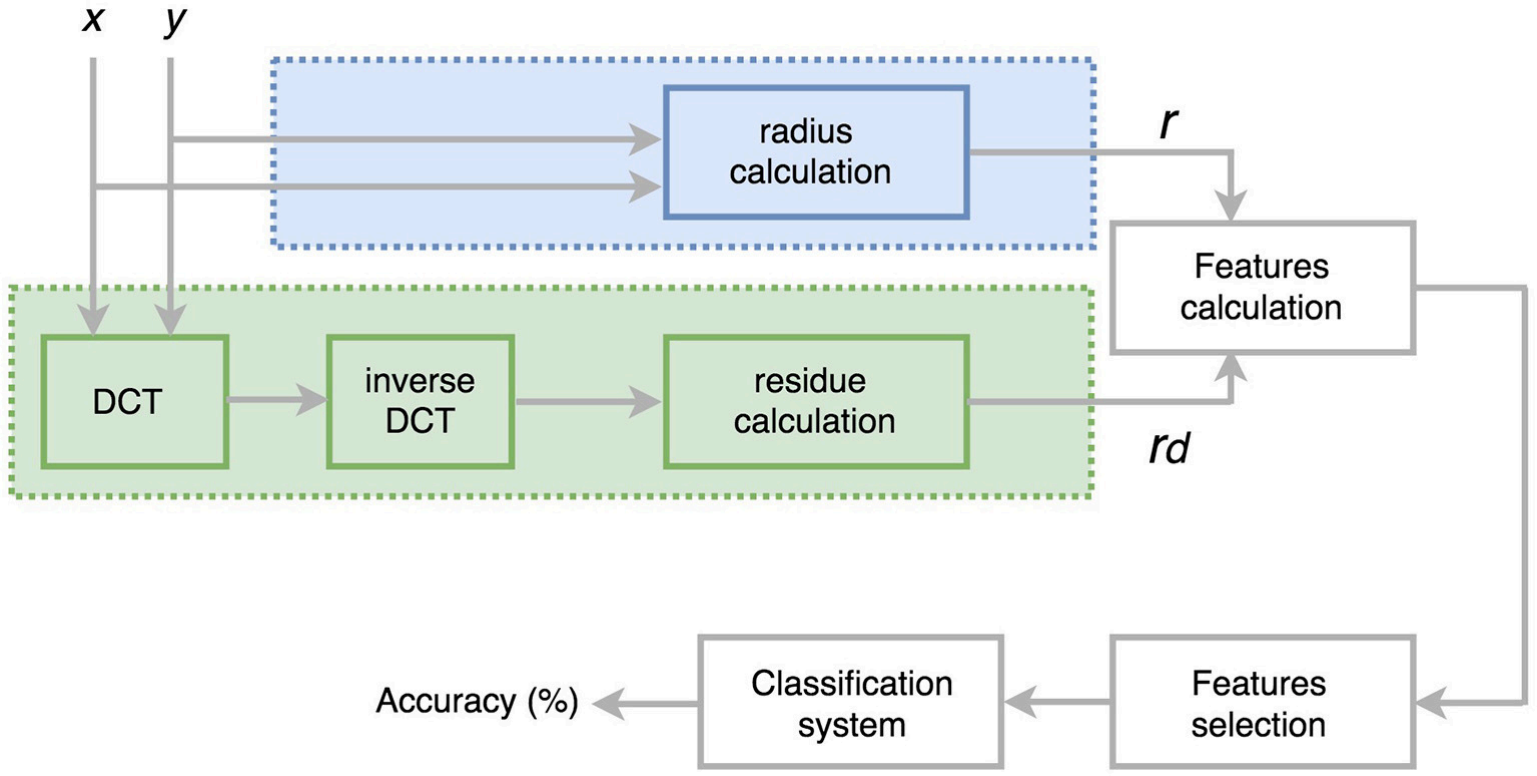
Block diagram of the experimental part.

**Figure 3:**
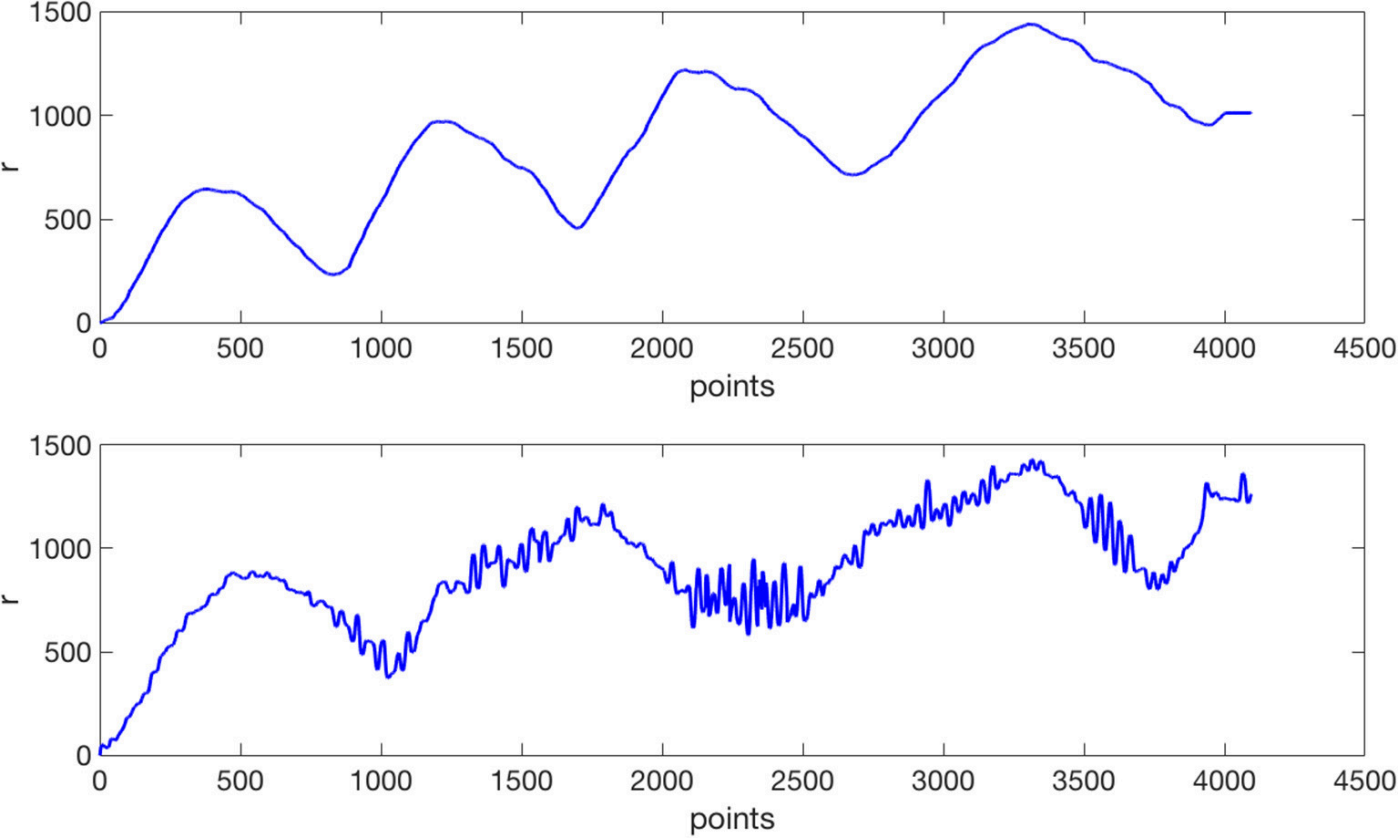
An example of Archimedes' spirals radius *r* performed by the same subjects of Figure 1. At the top for the control subject; at the bottom for the ET patient.

**Figure 4:**
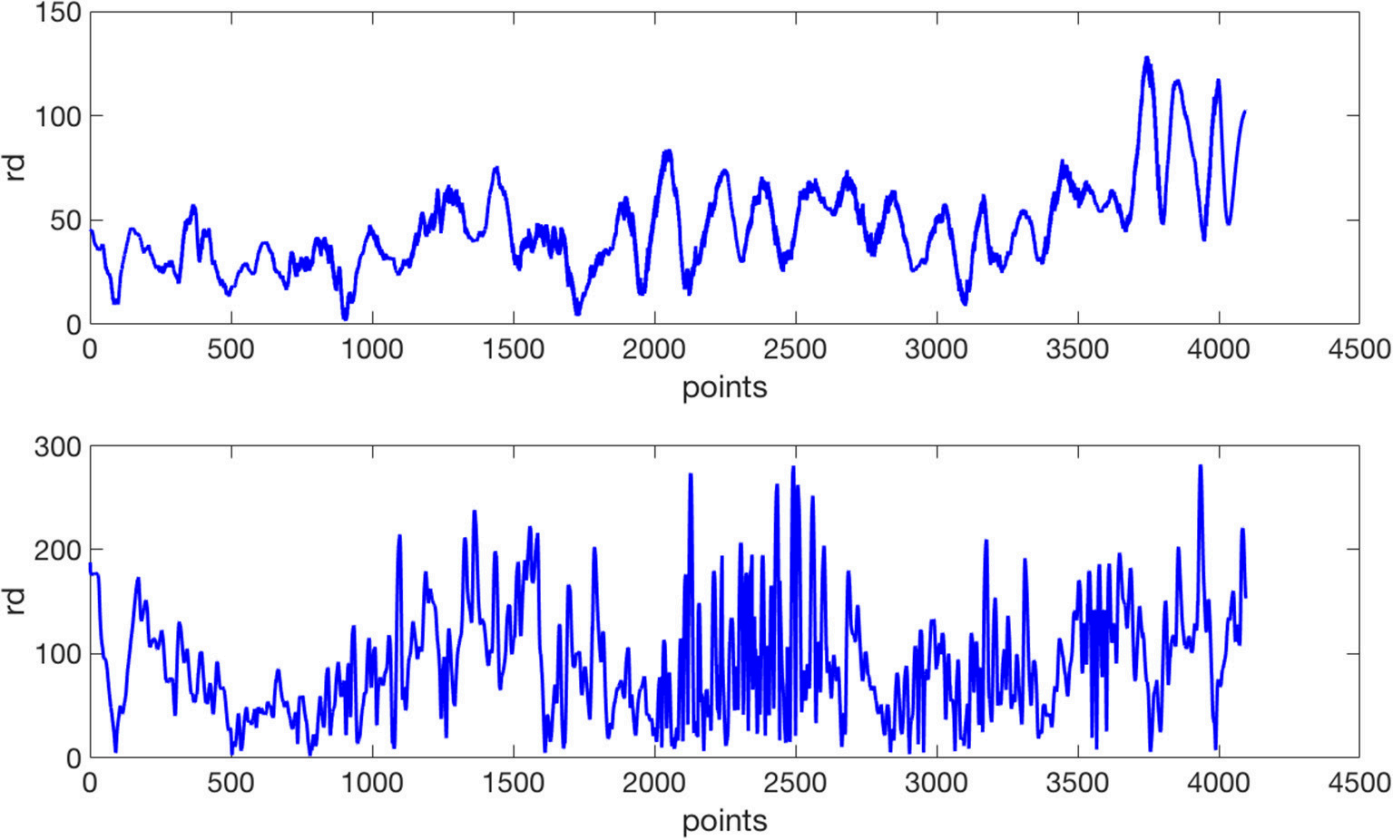
An example of the residue *rd* performed by the same subjects of Figure 1. At the top for the control subject; at the bottom for the ET patient.

To characterize each spiral by means of a single real sequence the spatial coordinates (*x*_*n*_, *y*_*n*_) can be combined in several ways. We investigate two options: (i) calculating the radius of the polar coordinates and (ii) estimating a distance. Figure 2 shows a block diagram of the two processes:

- The radius method: in this case, the radius was calculated by transforming the Cartesian coordinates to Polar coordinates. Therefore, the new sequence *r*_*n*_ was obtained as $r_{n}=\sqrt{{x_{n}}^{2}+{y_{n}}^{2}}$. An example of the radius sequence *r*_*n*_ for a healthy subject and a patient is shown in Figure 3.
- The residue method: in this case, the Cosine transform was applied to each coordinate *x*_*n*_and *y*_*n*_ separately, and then the inverse Cosine transform was calculated using a predefined number of coefficients, obtaining the estimated sets ${\tilde{x}}_{n}$ and ỹ_*n*_. The inverse Cosine Transform of each axis was subtracted from the original signal and we obtained the residue calculated as the distance between the two signals. Finally, we characterize each spiral with a single real sequence *rd*_*n*_ obtained from de residues ${\hat{x}}_{n}$ and ŷ_*n*_ of (*x*_*n*_, *y*_*n*_) as follows:
  (7) $$\begin{array}{l} rd_{n}=\sqrt{{\left(x_{n}-{\tilde{x}}_{n}\right)}^{2}+{\left(y_{n}-{ỹ}_{n}\right)}^{2}}=\sqrt{{\left({\hat{x}}_{n}\right)}^{2}+{\left({ŷ}_{n}\right)}^{2}} \end{array}$$
- An example of the sequences *rd*_*n*_ is shown in Figure 4. In order to evaluate the effect of the number of coefficients, several number of coefficients have been considered in the experiments.

By visual observation, comparing Figures 3, **4**, we notice that the irregularity of the signal is, as expected, bigger for the ET subjects compared to controls, and more notorious in residue than in the radius signal.

From these two signals, the radius and the residue, we extract a set of temporal and frequency features. The temporal features are, for example, the root mean square, standard deviation, maximum fractal length, or zero crossing. Frequency features, obtained from the Welch periodogram transform, are, for example, the mean frequency and its amplitude, median frequency, total power, 1st, 2nd, and 3rd spectral moments, kurtosis, or autocorrelation. The complete list of features is shown in Table 2 (temporal domain features) and Table 3 (frequency domain features). We refer the reader to Shair et al. (2017) for details on the features and how to calculate them. The total number of extracted features is 34 and we will use feature selection algorithm to keep the most discriminative ones.

**Table 2:** List of the extracted features from the temporal domain.

**Temporal Features**	**Descriptor**
Sample entropy (SENT)	*m* = 3, *r* = 0.2
Mean absolute value (MAV)	$\frac{1}{N}\sum_{i=1}^{N}|X_{i}|$
Variance (VAR)	$\frac{1}{N-1}\sum_{i=1}^{N}|X_{i}-\mu {|}^{2}$
Root mean square (RMS)	$\sum_{i=1}^{N}\frac{1}{N}X_{i}^{2}$
Log detector (LOG)	$e^{\frac{1}{N}\sum_{i=1}^{N}log\left(\left|X_{i}\right|\right)}$
Waveform length (WL)	$\sum_{i=1}^{N-1}|X_{i+1}-X_{i}|$
Standard deviation (STD)	$\sqrt{\frac{1}{N-1}\sum_{i=1}^{N}|X_{i}-\mu {|}^{2}}$
Difference Absolute standard deviation (AAC)	$\sqrt{\frac{1}{N-1}\sum_{i=1}^{N-1}{\left(X_{i+1}-X_{I}\right)}^{2}}$
Fractal dimension (FD)	Higuchi's algorithms with *m* = 5
Maximum fractal length (MFL)	$log\left(\sum_{i=1}^{N-1}|X_{i+1}-X_{i}|\right)$
Myopulse percentage rate (MYO)	Percentage of time where the signal is bigger than two times the mean
Integrated EMG (IEMG)	$\sum_{i=1}^{N}|X_{i}|$
Simple square EMG (SSI)	$\sum_{i=1}^{N}X_{i}^{2}$
Zero crossing (ZC)	The number of times in which the signal crosses its mean
Slope sign change (SSC)	The number of times in which the slope of the sign changes
Wilson amplitude (WAMP)	$\sum_{i=1}^{N-1}|X_{i}-\;X_{i+1}|>\epsilon$ where ϵ is the mean of the signal
Autoregressive coefficients (AR, 4 coefficients)	AR parameter estimation via Yule-Walker method

*The descriptor includes the values of the parameters (when needed) and/or the mathematical definition. Details on all the features can be found in Shair et al. (2017)*.

**Table 3:** List of the extracted features from the frequency domain.

**Frequency Features**	**Descriptor**
Main peak amplitude (Pmax)	Maximum peak
Main peak frequency (Fmax)	Frequency of the max peak
Mean power (MP)	$\frac{1}{N}\sum_{i=1}^{N}|P_{i}|$
Total power (TP)	$\sum_{i=1}^{N}P_{i}$
Mean frequency (MNF)	Estimates the mean normalized frequency of the power spectrum
Median frequency (MDF)	Estimates the median normalized frequency of the power spectrum
Standard deviation (STD)	$\sqrt{\frac{1}{N-1}\sum_{i=1}^{N}|P_{i}-\mu {|}^{2}}$
1st spectral moment (SM1)	Spectral moments
2nd spectral moment (SM2)	Spectral moments
3rd spectral moment (SM3)	Spectral moments
Kurtosis (KUR)	Kurtosis of the power spectrum
Skewness (SKW)	Skewness of the power spectrum
Autocorrelation (Auto, 3 coefficients)	3 firsts coefficients of the autocorrelation

*The descriptor includes the values of the parameters (when needed) and/or the mathematical definition. Details on all the features can be found in Shair et al. (2017)*.

### Classification Systems

Linear Discriminant analysis (LDA), k-nearest neighbors (k-NN) and support vector machine (SVM) with radial basis kernel have been used as classification algorithm to discriminate between ET and control subjects. To evaluate the performance of these algorithms we implemented the leave-one-out technique. Although all the drawing samples have been done with a template and the same pen tablet, the number of drawing points acquired was different for each sample. In order to ensure the same number of points in each sequence, we resampled all the exercises to enforce 4096 points in all of them. Establishing the same number of points is mandatory in order to be able to compare the different Cosine transforms. Normalization was also applied in order to have a unit norm in all the features. Results were evaluated by means of the Accuracy (%). In the training and validation steps we use a *k*-fold cross validation strategy with *k* = 10. Cross-validation is a robust technique for the selection of variables and widely used to obtain realistic results reducing overfitting.

### Experiments

Experiments where carried on the BIODARWO dataset. A feature selection algorithm was applied in order to improve the classification rate removing the similarities and dependencies between features. Relieff algorithm (Kononenko et al., 1997) was selected for its well performance in binary classification problems. This method is one of the best enabling the classifiers to achieve the highest classification accuracy while reducing the number of unnecessary attributes. Also, and very important for us, Relieff gives as output an ordered list of features according to their importance, which will allow us to select the first of them (Molina et al., 2002; Cehovin and Zoran, 2010). In this study the Relieff algorithm implementation from MATLAB.

The feature selection algorithm was applied to the residue and radius features in order to obtain the best performance in both cases. Several numbers of features were tested and experimentally we obtained the best performance using the top 5-predictor rank features. The 5 characteristics selected in each case are the following, sorted according to their importance:

Residue method:
  1. Mean frequency (MNF)
  2. Wilson amplitude (WAMP)
  3. Mean absolute value (MAV)
  4. Maximum fractal length (MFL)
  5. Fractal dimension (FD)
Radius method:
  1. Maximum fractal length (MFL)
  2. Fractal dimension (FD)
  3. Myopulse percentage rate (MYO)
  4. Mean absolute value (MAV)
  5. Standard deviation (STD)

## Results and Discussion

Three different classification algorithms have been used to compare the performance of the residue method and the radius method. For the residue method, several coefficients for the inverse cosine transform were considered in order to establish the optimal value.

First, a LDA was used. As can be seen in Table 4, the maximum accuracy was 85.71% obtained for the residue method with 17 coefficients, while for the radius method the best accuracy was 75.51%. An improvement of 10% was achieved using the cosine transform apporach, instead of working directly with the radius. This emphasizes the importance of using the residue as a time series rather than working directly with the radius, as the residue contains more information regarding the tremor. We can see the results of the LDA as a reference results, and the other systems will try to improve these ones.

**Table 4:** Accuracy (%) for the LDA classifier for the residue of the cosine transform (as a function of the number of coefficients considered) and for the radius.

	**Coefficients**											
	**10**	**15**	**16**	**17**	**18**	**20**	**21**	**22**	**23**	**25**	**30**	**50**
Residue of the CT	75.51	79.59	81.63	**85.71**	79.59	71.43	77.55	79.59	77.55	79.59	77.55	77.55
Radius	75.51											

The best result is highligted in bold

Next, the k-NN method was used. In this case, different number of neighbors were tested. Results are shown in Table 5 for the residue method, and in Table 6 for the radius method. The maximum accuracy was 83.67% obtained for the residue method with 17 coefficients and 3 neighbors, while for the radius method the best accuracy was 81.63% with 3, 4, and 5 neigbors. We note that results are worst than the ones obtained using LDA, but again, the residue method outperforms the radius method, even if that now the difference is smaller. The number of neighbors can be kept small (in both cases 3 was enough), which is interesting from the point of view of simplicity. The number of coefficients for the inverse cosine transform was again 17.

**Table 5:** Accuracy (%) from k-NN classifier and residue method, where k stands for the number of neighbors used in the classification algorithm.

	**Coefficients**											
***k***	**10**	**15**	**16**	**17**	**18**	**20**	**21**	**22**	**23**	**25**	**30**	**50**
1	75.51	75.51	77.55	79.59	77.55	67.34	75.51	75.51	77.55	73.46	73.46	77.55
3	69.38	77.55	73.46	**83.67**	77.55	65.30	71.42	73.46	67.34	65.30	69.38	63.26
5	69.38	79.59	71.42	77.55	73.46	59.18	77.55	71.42	69.38	65.30	69.38	67.34
7	73.46	73.46	73.46	79.59	81.63	63.26	69.38	67.34	71.42	71.42	67.34	69.38
9	77.55	75.51	73.46	73.46	69.38	73.46	67.34	65.30	69.38	69.38	67.34	73.46
11	77.55	77.55	77.55	77.55	75.51	69.38	71.42	63.26	67.34	63.26	59.18	79.59
13	77.55	77.55	73.46	73.46	67.34	65.30	71.42	61.22	65.30	65.30	69.38	79.59
15	77.55	75.51	71.42	73.46	69.38	69.38	71.42	67.34	67.34	67.34	69.38	77.55
17	71.42	75.51	69.38	79.59	73.46	69.38	71.42	63.26	65.30	69.38	69.38	73.46
19	71.42	75.51	71.42	81.63	77.55	67.34	71.42	63.26	61.22	65.30	63.26	67.34
21	69.38	75.51	73.46	79.59	75.51	71.42	71.42	75.51	65.30	61.22	61.22	71.42
23	73.46	75.51	73.46	79.59	73.46	75.51	71.42	73.46	75.51	71.42	67.34	69.38
25	67.34	77.55	69.38	77.55	77.55	69.38	73.46	73.46	65.30	67.34	71.42	73.46
27	67.34	77.55	71.42	73.46	73.46	67.34	73.46	75.51	67.34	73.46	73.46	75.51
29	69.38	67.34	73.46	75.51	73.46	71.42	73.46	71.42	69.38	69.38	71.42	73.46
31	71.42	67.34	71.42	75.51	73.46	71.42	73.46	71.42	71.42	67.34	69.38	69.38
33	71.42	73.46	67.34	71.42	71.42	67.34	71.42	71.42	73.46	69.38	69.38	75.51

*The best result is highligted in bold*.

**Table 6:** Accuracy (%) from k-NN classifier and radius features, where k stands for the number of neighbors used in the algorithm.

**k**	**CR(%)**
1	77.55
3	**81.63**
5	**81.63**
7	**81.63**
9	79.59
11	77.55
13	77.55
15	73.46
17	69.38
19	69.38
21	69.38
23	67.34
25	67.34
27	65.30
29	65.30
31	63.26
33	61.22

The best results is highligted in bold

Then, we used explored a non-linear classification system. Specifically we used an SVM with RBF kernel. The number of coefficients of the inverse cosine transform was explored and, as in the other two cases, we found that 17 was the best case. Therefore, we established 17 coefficients and then we performed a tunning for the kernel scale and penalty cost of missclassifaction in order to achieve the best classification rate (accuracy). Results for the residue method with 17 coefficients are presented in Table 7. The maximum accuracy achieved with this approcah was 95.92%, for the scale of 0.2 and costs 10^3^ and 10^4^. Several other combinations reached accuracies over 90%, which is a very good result. For the radius method, results are shown in Table 8. In this case the maximum accuracy was 85.71%, for the cost 10^4^ and scales 0.7–1. This result outperforms the ones obtained previously with LDA and k-NN.

**Table 7:** Accuracy (%) from SVM RBF classifier and residue features with 17 coefficients, where cost stands for the penalty cost of missclassification and scale is the kernel scale applied.

	**Scale**										
**Cost**	**0.1**	**0.2**	**0.3**	**0.4**	**0.5**	**0.6**	**0.7**	**0.8**	**0.9**	**1**	**1.1**
10^−5^	55.10	55.10	55.10	55.10	55.10	55.10	55.10	55.10	55.10	55.10	55.10
10^−4^	55.10	55.10	55.10	55.10	55.10	55.10	55.10	55.10	55.10	55.10	55.10
10^−3^	55.10	55.10	55.10	55.10	55.10	55.10	55.10	55.10	55.10	55.10	55.10
10^−2^	55.10	55.10	55.10	55.10	55.10	55.10	55.10	55.10	55.10	55.10	55.10
10^−1^	55.10	65.31	55.10	55.10	55.10	55.10	55.10	55.10	55.10	55.10	55.10
1	85.71	81.63	77.55	77.55	77.55	79.59	77.55	75.51	73.47	69.39	61.22
10^1^	89.80	87.76	87.76	89.80	81.63	81.63	81.63	79.59	79.59	79.59	79.59
10^2^	91.84	93.88	89.80	87.76	87.76	89.80	89.80	89.80	89.80	89.80	89.80
10^3^	91.84	**95.92**	91.84	83.67	87.76	89.80	85.71	89.80	87.76	87.76	87.76
10^4^	91.84	**95.92**	91.84	91.84	91.84	87.76	83.67	85.71	87.76	87.76	87.76

*The best result is highligted in bold*.

**Table 8:** Accuracy (%) from SVM with RBF kernel classifier and radius features, where cost stands for the penalty cost of missclassification and scale is the kernel scale applied.

	**Scale**										
**Cost**	**0.1**	**0.2**	**0.3**	**0.4**	**0.5**	**0.6**	**0.7**	**0.8**	**0.9**	**1**	**1.1**
10^−5^	55.10	55.10	55.10	55.10	55.10	55.10	55.10	55.10	55.10	55.10	55.10
10^−4^	55.10	55.10	55.10	55.10	55.10	55.10	55.10	55.10	55.10	55.10	55.10
10^−3^	55.10	55.10	55.10	55.10	55.10	55.10	55.10	55.10	55.10	55.10	55.10
10^−2^	55.10	55.10	55.10	55.10	55.10	55.10	55.10	55.10	55.10	55.10	55.10
10^−1^	67.35	63.27	55.10	55.10	55.10	55.10	55.10	55.10	55.10	55.10	55.10
1	77.55	79.59	77.55	77.55	77.55	75.51	73.47	73.47	69.39	63.27	61.22
10^1^	75.51	77.55	79.59	81.63	79.59	79.59	77.55	77.55	77.55	77.55	77.55
10^2^	71.43	73.47	81.63	77.55	77.55	81.63	79.59	79.59	79.59	79.59	79.59
10^3^	67.35	71.43	79.59	83.67	83.67	81.63	79.59	77.55	75.51	75.51	77.55
10^4^	63.27	75.51	75.51	79.59	81.63	83.67	**85.71**	**85.71**	**85.71**	**85.71**	83.67

*The best result is highligted in bold*.

In order to demonstrate the capability of the system, Table 9 (left) presents the confusion matrix obtained with the residue method, for 5 features and the SVM classifier. From these values we can calculate the sensitivity (SEN) and specificity (SPE) of the system, which results in the following values:

(8) $$\begin{array}{l} SEN=\frac{TP}{TP+FN}=\frac{20}{21}=95.24\;\%\\ SPE=\frac{TN}{TN+FP}=\frac{26}{28}=92,86\;\% \end{array}$$

where TP, TN, FP, and FN stands for the true positive, true negative, false positive and false negative values of the confusion matrix.

**Table 9:** Confusion matrix obtained when using a SVM classifier.

		**Predicted**	
		**ET**	**Control**
Actual	ET	20	1
	Control	2	26
Actual	ET	21	0
	Control	1	27

*On the top, with the residue method (5 features); on the bottom with the residue method plus 2 features of the radius method: Maximum fractal length and Fractal dimension*.

Finally, we explore the combination of both methods (residue and radius features). For that, we started from the best previous case (SVM with RBF, using the 5 features of the residue method) and adding 1 feature of the radius method; then adding 2 features; then 3 features; then 4 features and finally the 5 features. When adding new features, we followed the ranking presented in section 2.6. For the case of 5 (residue) + 2 (radius) features (see Table 10) we achieved an accuracy of 97.96%, outperforming the best result obtained before. The radius features added that contributed to increase the accuracy were the Maximum fractal length and the Fractal dimension. In that case, the confusion matrix (see Table 9) contains only one missclassified sample, which corresponds to a control subject that the system classifies as ET. Therefore, sensitivity and specificity are increased to the following values: *SEN* = 100%; *SPE* = 96.42%, see (9). The exact same result whas obtained for 5(residue) + 3(radius), in that case adding also the Myopulse percentage ratio.

(9) $$\begin{array}{l} SEN=\frac{TP}{TP+FN}=\frac{21}{21}=100\;\%\;\\ SPE=\frac{TN}{TN+FP}=\frac{27}{28}=96,42\;\% \end{array}$$

We can see that for the three classification systems the residue method always obtained the best accuracies. In particular, the SVM classifier was the best choice for both methods, and the results obtained with the residue method clearly outperforms the results obtained with the radius method. The best results, using only one of the methods is close to 96% of accuracy, clearly exceeding the best results obtained in (Lopez De Ipina et al., 2015) and (López-de-Ipiña et al., 2018) and similar to those obtained in (López-de-Ipiña et al., 2016), in all the cases using the same database. But the combination of both methods allowed to increase up to almost 98% of accuracy. This is interesting because it means that some information is complementary and therefore useful for the classifier.

**Table 10:** Accuracy (%) from SVM with RBF kernel classifier, residue features plus the following radius features: Maximum fractal length and Fractal dimension.

	**Scale**										
**Cost**	**0.1**	**0.2**	**0.3**	**0.4**	**0.5**	**0.6**	**0.7**	**0.8**	**0.9**	**1**	**1.1**
10^−5^	55.1	55.1	55.1	55.1	55.1	55.1	55.1	55.1	55.1	55.1	55.1
10^−4^	55.1	55.1	55.1	55.1	55.1	55.1	55.1	55.1	55.1	55.1	55.1
10^−3^	55.1	55.1	55.1	55.1	55.1	55.1	55.1	55.1	55.1	55.1	55.1
10^−2^	55.1	55.1	55.1	55.1	55.1	55.1	55.1	55.1	55.1	55.1	55.1
10^−1^	55.1	65.31	55.1	55.1	55.1	55.1	55.1	55.1	55.1	55.1	55.1
1	87.76	81.63	81.63	79.59	79.59	77.55	79.59	79.59	73.47	67.35	67.35
10^1^	**97.96**	89.8	89.8	83.67	83.67	83.67	81.63	81.63	81.63	81.63	81.63
10^2^	**97.96**	95.92	93.88	91.84	91.84	89.8	91.84	89.8	89.8	89.8	83.67
10^3^	**97.96**	95.92	93.88	91.84	93.88	91.84	91.84	91.84	93.88	93.88	93.88
10^4^	**97.96**	95.92	93.88	91.84	93.88	91.84	91.84	91.84	89.8	89.8	89.8

*The best result is highligted in bold. Cost stands for the penalty cost of missclassification and scale is the kernel scale applied*.

It is important to emphasize that while all the possible characteristics captured by the Intuos device (including pressure, air time, surface time, azimuth and elevation angles, speed, acceleration, etc.), were used in the previous works, now only the *x* and *y* coordinate points are used. For example, comparing our results with those presented in our recently publiched work (López-de-Ipiña et al., 2018), we can see that we propose a new set of extremely reduced features derived directly from the *x* and *y* coordinate points, which allows us to obtain better results (97.96% against 91%) than those in (López-de-Ipiña et al., 2018). We combined *x* and *y* coordinate values in two ways: (i) calculating the radius and (ii) calculating the residue after reconstructing the coordinate points using the cosine and inverse cosine transforms. With only this information we were able to outperform the best accuracy obtained in previous results, with a very simple method and using only 7 features, instead of 70 to 198 features used in (López-de-Ipiña et al., 2016), for example.

The results of our study will allow its implementation in real time by means of a validation study to confirm its usefulness in the differential diagnosis of essential tremor with respect to other entities with which it can be confused such as physiological tremor, tremor in Parkinson's disease and dystonia, as well as in the evolutionary monitoring of essential tremor after the start of any of the specific treatments already available.

There are several reasons to explore only these features. Among them, the simplicity to obtain them, because this is the traditional available information of any acquiring system. This can make it easier and allow the use of other simpler acquisition systems by tracking only the *x* and *y* coordinate values, rather than, for example, the pencil angles or the pressure exerted during the drawing process. Then, using fewer phisical variables will lead to a small number of features and therefore also simple clasification methods. Finally, the computational time is also affected by the simplicity or complexity of the data to acquire, the features to extract and the clasification system to implement. Using only information of the *x* and *y* coordinates allowed us to reduce complexity and hence also computational time. This is important if we want to work in real time in autonomous systems, which is one of the goals of the abovementioned project.

Working with the residue is clearly a good option, outperforming in all the cases the results obtained directly with the radius, and the small set of features, all of them that can be interpreted by health specialists in order to investigate the relevance and usefulness of the biomarkers for early diagnosis of ET.

## Conclusions

Nowadays, a large number of models of wireless triaxial accelerometers and gyroscopes that allow clinical assessment of postural and kinetic tremor are In addition, there are new techniques in development such as the measurement of the components of recovery of the blinking reflex, kinematic measurements, analysis of accelerometry data and computerized measurements of the ocular movement whose objective is to be able to objectively distinguish the physiological tremor from the essential tremor, mainly when it is of mild severity. Unfortunately, the advantages of high sensitivity and accuracy in the linear register of portable motion transducers are mitigated by the large variability in the random amplitude of the tremor.

This work analyzed the capability of the cosine transform as a technique to be used for obtaining relevant biomarkers from drawings and handwriting. This is part of a wider cross study on the diagnosis of essential tremor, which is developed in the Biodonostia Health Institute. Specifically, the main goal was to obtain good results using simple information provided by the *x* and *y* coordinates of the Archimedes' spiral drawing. The collection of a standardized writing sample is a method used in clinical practice and research to assess the severity of tremor. The method has many practical advantages. It is easy to obtain and takes little time to implement. In fact, the samples can even be collected remotely using different devices, allowing them to be studied in different real-life situations and saving time and resources when it comes to evaluating a large number of people. Surprisingly, there are virtually no published data to address a methodological problem that arises: the validity of the method. The performance of the hand-drawn spiral as a screening tool for Essential Tremor depends to some extent on the sample of case studies and the cut-off points used for sensitivity and specificity.

We investigated two possibilities, the first one using the radius derived directly by transforming the Cartesian coordinates to Polar coordinates, and the second one using the residue calculated as the distance between the coordinates and its reconstruction by means of the pair cosine transform / inverse cosine transform at a given number of the coefficients. Classical features, both temporal and frequential, were derived for both cases and the Relieff method was used to reduce the set to the top 5-predictor rank features. Interestingly, 3 of the 5 features are common in both cases. Also, notice that for the radius method all the features are from time domain while for the residue method, one is from frequency domain and the others from time domain. This seems to point out that time domain features are very relevant. The results using only one of the methods are optimal for the residue method, with accuracy up to almost 96% using only 5 features using a SVM classification system. But the results are even better (only one misclassified sample) when adding the first two features of the radius method (fractal related), reaching an accuracy of almost 98% with 7 features.

Louis (2015) demonstrated that the hand-drawn spiral is a sensitive and specific screening method as a measure of tremor severity for those tremors of mild to moderate amplitude or greater according to the WHIGET Tremor Rating Scale. This scale allows to rate postural and kinetic tremor during each test, including the four hand-drawn spirals: 0 (none), 1 (mild), 2 (moderate), 3 (severe). In his study, when the tremor ratio was ≥1.5 (i.e., a mild to moderate tremor) the spiral analysis obtained a sensitivity between 78.8 and 97.0% depending on the samples and a specificity of 95.3%. The analysis proposed in our work achieves 100% sensitivity and 96.42% specificity even when the severity cut-off point is reduced to mild amplitude tremors (which would correspond to 1 on the WHIGET scale). In fact, the only badly classified case of our sample corresponds to a control without TE that has oscillations in the trace and that corresponds to a case of exaggerated physiological tremor (due to stress, drug consumption, etc.) and that can be classified as <1 in the scale. At present, these cases can only be adequately discriminated by means of more sophisticated tests such as the electromyographic register. Therefore, our method significantly improves the analysis of drawing results as a tremor screening tool because it allows for the proper classification of almost all tremor cases, even those of slight amplitude. This is interesting for real-time applications, because the computational cost is very low. Given the interesting results obtained by the cosine transform applied to the *x* and *y* coordinates, in future works we will evaluate this transform on the pressure and other direct characteristics measured by the digitation tablet.

## Ethics Statement

This study was carried out in accordance with the recommendations of the Ethics Committee of the Donostia University Hospital (San Sebastian, Spain), which approved the protocol. All subjects gave written informed consent in accordance with the Declaration of Helsinki.

## Author Contributions

JS-C, PM-P, and KL conceived the algorithm. IA-E, PM-P, and JS-C implemented the feature selection algorithm and the classification algorithm and performed the data analysis. PC and IS-M collected the experimental data, and contributed to the signal processing section. KL and AB had theoretical contributions on the analysis of the results. JS-C, IA-E, KL, and PC wrote the first draft of the paper. All authors reviewed the draft of the paper and approved the final manuscript.

### Conflict of Interest Statement

IA-E was employed by company Seidor Labs as a PhD student. The remaining authors declare that the research was conducted in the absence of any commercial or financial relationships that could be construed as a potential conflict of interest.
